# Quantitative metrics and psychometric scales in the visual art and medical education literature: a narrative review

**DOI:** 10.1080/10872981.2021.2010299

**Published:** 2021-12-05

**Authors:** John David Ike, Joel Howell

**Affiliations:** a Clinical Scholar, National Clinician Scholars Program, Clinical Instructor, Division of Hospital Medicine, Hospitalist, Ann Arbor Va Healthcare System, University of Michigan, Ann Arbor; b Elizabeth Farrand Collegiate Professor of the History of Medicine, Professor, Departments of Medicine and History, University of Michigan, Ann Arbor

**Keywords:** Medical education, medical humanities, curriculum development, visual arts, observation skill

## Abstract

The authors conduct a narrative review of the quantitative observation metrics and psychometric scales utilized in the visual arts and medical education literature in order to provide medical educators with a ‘toolkit’ of quantitative metrics with which to design and evaluate novel visual arts-based pedagogies. These efforts are intended to support the AAMC and National Academy of Sciences, Engineering, and Medicine’s aims to formally evaluate and integrate arts and humanities curricula into traditional scientific educational programming. The scales reviewed examine a variety of domains including tolerance for ambiguity, bias, burnout, communication, empathy, grit, and mindfulness/reflection. Observation skill, given the heterogeneity of quantitative metrics, is reviewed separately.

## Introduction

Since medical humanities’ inception in the 1960s, it has grown to encompass a wide range of disciplines, including literature, narrative medicine, music, dance, and others. As of 2018, roughly 85% of medical educational institutions offer some programming in the medical humanities[1]. Starting in the late 1990s, educators began exploring the efficacy of visual arts programming for improving the ability of medical trainees to deliver high-quality healthcare. Most initial studies examined the relationship between visual art programming and skill at visual observation [2,3]. In the years that followed, educators broadened their conception of the potential value of visual arts-based curricula. Some hoped these curricula could help stem rising rates of burnout, others were concerned about changes in medical student empathy, others about tolerance for ambiguity [4–11]. Many educators hoped to demonstrate the value of such training. But how, exactly, does one compute the full impact of engaging with a work of visual art? Educators have attempted to answer this and similar questions by evaluating visual arts programming with a range of tools both qualitative and quantitative [12–16].

Meanwhile, attention to arts and humanities programming by major professional organizations has increased. In 2018, the National Academy of Sciences, Engineering, and Medicine (NASEM) encouraged a renewed engagement with the arts and humanities in all educational pedagogy[17]. In 2020, the Association of American Medical Colleges (AAMC) called for the formal integration of the arts and humanities into healthcare education and crafted a digital ‘Getting Started’ guide [18,19]. The AAMC also endorsed a framework for developing medical humanities curricula, the Prism model [20,21]. Both the AAMC and NASEM advocated for novel pedagogical approaches, enhanced research, and a need to measure ‘learner outcomes beyond satisfaction with the course or program.’[18]

Some medical humanities educators argue that quantitative measurement of the effects of visual arts-based programming is unnecessary [16,22,23]. But curricular committees may be hesitant to add new medical humanities courses into a crowded curriculum without some form of outcomes-based assessment. While acknowledging the value of qualitative forms of analysis, many leaders in clinical schools may be more comfortable if arguments for visual arts integration are accompanied by some quantitative analysis. As the field matures, scholars may want to compare the impact of curricular programming across different institutions, a task that requires consistent metrics for evaluation. We offer a narrative review of the quantitative observation metrics and psychometric scales utilized in the visual arts and medical education literature in order to provide medical educators with a ‘toolkit’ of quantitative metrics with which to design and evaluate novel visual arts-based pedagogies.

## Methods – Narrative Review

We conducted an exploratory search of MEDLINE, Web of Science, and Google Scholar for studies that evaluated the role of visual arts in medical education. The search was done in September 2020 with assistance from a health services research librarian. Summative reports, review articles, and bibliographies related to the visual arts and medical education were also reviewed [12–18]. A repeat search of MEDLINE utilizing identical search terms was conducted in April 2021. Inclusion criteria were limited to visual arts-based studies that incorporated quantitative outcomes metrics, were written in English, and conducted in USA and Canadian medical schools or graduate medical education training programs. Studies written before 1980 were excluded. These searches yielded fourteen visual arts and medical education studies [3,24–36]. Strictly qualitative studies are well-summarized elsewhere and were not included in this study [13,15]. This literature review was conducted by one author (JDI) with oversight by the second author (JDH). Below we first address observation and physical diagnosis metrics and then describe the remaining psychometric scales alphabetically (Table 1, Table 2). We do not offer a detailed critique of each metric’s design and validation in this narrative review; our intent is to make authors aware of possible assessment techniques as well as their global benefits and limitations. Moreover, as our narrative review of quantitative and psychometric measurements is limited to those used in the visual arts and medical education literature, there are likely other scales that are not explored in this review.

**Table 1.** Observation Scales and Measurements used to Assess Visual Arts Programming

Quantitative Observation Metrics	Visual Arts-Based Studies
Number of observations(± prespecified grading rubric) Time spent observing an image Number of words used to describe an image	(1) Dolev et al. 2001 – Pre-/post-testanalysis of clinical images gradedon a 9 or 10-point rubric. (2) Naghshineh et al. 2008 – Pre-/post-test analysis of patient photographs and artistic images usinga prespecified rubric. A qualitativeanalysis of image descriptions wasalso undertaken. (3) Klugman et al. 2011 – Pre-/post-testanalysis of clinical and artisticimages by word count and numberof observations. (4) Jasani et al. 2013 – Pre-/post-testanalysis of patient photographs bynumber of observations.A qualitative analysis of imagedescriptions was also undertaken. (5) Klugman et al. 2014 – Pre-/post-testanalysis of clinical images by wordcount and number of observations. (6) Huang et al. 2016 – Pre-/post-testanalysis of artistic and dermatologicimages by number of observations. (7) Gurwin et al. 2017 – Pre-/post-testanalysis of clinical and visual artimages. Clinical images were gradedon a prespecified rubric. Visual artimages were subjectively awardedpoints for observations,associations,interpretations, flexibility, andempathizing (no prespecifiedrubric). (8) Goodman et al. 2017 – Pre-/post-test analysis of radiographic imagesgraded on prespecified rubrics. (9) Agarwal et al. 2020 – Pre-/post-testanalysis of clinical images by wordcount and time spent analyzing each image. A qualitativeanalysis of image descriptions was alsounderta ken.

**Table 2.** Psychometric Scales Utilized to Assess Visual Arts Programming (All domains were evaluated using Likert Scales)

Domain	Psychometric Scale	Means Assessed	Visual Arts-based Studies Utilizing Scale(s)
**Ambiguity**	Modified Tolerance for Ambiguity Scale (TFA)	A 7-item questionnaire that measures an individual’s comfort (or discomfort) with ambiguity.	Klugman et al. 2011 Klugman et al. 2014 Gowda et al. 2018 Strohbehn et al. 2020
**Bias**	Best Intentions Questionnaire (BIQ)	A 24-item questionnaire test that assesses how physician biases may affect patient care.	Gowda et al. 2018
**Burnout**	The Maslach Burnout Inventory (MBI)	A 22-item questionnaire that measures three components of burnout in health professional populations: emotional exhaustion, depersonalization, and low sense of personal accomplishment.	Orr et al. 2019
**Communication**	Communication Skills Attitudes Scale (CSAS)	A 26-item questionnaire that assesses student perceptions towards learning communication skills.	Klugman et al. 2011 Klugman et al. 2014
**Empathy & Compassion**	The Compassion Scale Inter-reactivity Index (IRI) Jefferson Scale of Empathy Reading the Mind in the Eyes	A 24-item questionnaire that measures compassionate responses through three conflicting domains: kindness-versus-indifferences, common humanity-versus-separation, and mindfulness-versus-disengagement. A 28-item questionnaire that measures affective empathy (empathic concern, personal distress) and cognitive empathy (perspective taking, fantasy). A 20-item questionnaire that measures cognitive and affective components of empathy in health professional populations. Theory of the Mind inspired scale designed to gauge an individual’s ability to intuit the emotional state of others through pairing an image of someone’s eyes with their internal emotional state.	Zazulak et al. 2015 – IRI scale Zazulak et al. 2017 – IRI scale, Compassion Scale Gurwin et al. 2017 – Reading the Mind in the Eyes Strohbehn et al. 2020 – JSPE
**Grit**	Short Grit Scale (GRIT-S)	An 8-item questionnaire that measures two components of grit: perseverance of effort and consistency of interest.	Strohbehn et al. 2020
**Mindfulness & Reflection**	The Five-Facet Mindfulness Scale The Groningen Reflection Ability Scale (GRAS) The Mindful Attention Awareness Scale (MAAS)	A 39-tem questionnaire that assesses five domains of mindfulness: description, self-expression, acts of self-awareness, non-judgment of inner experience, and non-reactivity to inner experience. A 23-item questionnaire that measures reflective ability along three domains: self-reflection, empathic reflection, and reflective communication. A 15-item questionnaire that assesses dispositional mindfulness, which is the ability to be aware of and pay attention to the present moment.	Zazulak et al. 2017 – Five Facet Mindfulness Scale Gowda et al. 2018 – GRAS Strohbehn et al 2020 – MAAS

## Visual Arts Programming and Medical Education – What Do We Measure?

### OBSERVATION AND PHYSICAL DIAGNOSIS

Observation skill is the most frequently assessed quantitative domain in the visual arts and medical education literature (Table 1) [3,25,26,28–33]. Such exploration is logical in that the practice of clinical medicine typically involves practitioners carefully observing patients’ bodies. However, as there are no established scales for measuring the accuracy of clinical observation, observation is typically assessed with de novo designed quantitative metrics. These scales often quantify multiple measurements including the number of distinct observations, the number of words used to describe clinical and artistic images, the amount of time spent analyzing an image, or the number of clinically or artistically relevant observations. However, such scales often involve a fair amount of inherent individual judgment – what, precisely, is a clinically or artistically relevant observation? Heterogeneity between quantitative observation skill metrics has limited inter-study comparison.

## AMBIGUITY

### The Modified Tolerance for Ambiguity Scale

The Budner Tolerance for Ambiguity (TFA) scale was designed in 1962 and later modified in 1993 for a medical audience by Gail Geller, a professor at Johns Hopkins School of Public Health[6]. The 7-item questionnaire is graded on a Likert Scale and measures an individual’s comfort (or discomfort) with ambiguity. Example statements include, ‘It really disturbs me when I am unable to follow another person’s train of thought’ and ‘A good task is one in which what is to be done and how it is to be done are always clear.’[6]

The TFA scale is included in the AAMC Matriculating Student Questionnaire and Graduation Questionnaire administered to all incoming and graduating medical students in the USA[8]. Intolerance for ambiguity has been found to be associated with increased psychological distress and reduced clinical performance [6,8,37,38]. Given the importance of working comfortably in what can often be a fluid and ambiguous clinical world, and considering the ambiguity intrinsic to many works of visual art, the TFA scale has been utilized in several visual art and medical education studies [24,27,31,32].

## BIAS

### Best Intentions Questionnaire

The Best Intentions Questionnaire (BIQ) was designed in 2010 for healthcare trainees by Anne Gill, a Doctor of Nursing in the Department of Pediatrics at Baylor[39]. The 24-item questionnaire is scored on several Likert Scales and assesses an individual’s understanding of their own biases. The first set of statements explores a participant’s perception of how their biases may impact clinical decision making with statements such as ‘physicians can have biases about patients about which they are unaware.’[39] The second half of the questionnaire probes if individuals believe they can learn to become aware of, manage, and eliminate their own biases. Lastly, participants are asked about their ability to recognize their own emotional state in addition to the emotional state of others. One visual arts and medical education study has used the BIQ metric[27].

## BURNOUT

### Maslach Burnout Inventory

The Maslach Burnout Inventory (MBI) was developed in 1981 by University of California Berkley psychologist Christina Maslach to measure burnout in a variety of professional groups[40]. The 22-item questionnaire is scored on a Likert Scale and evaluates three domains of burnout: emotional exhaustion (EE), depersonalization (DP), and sense of personal accomplishment (PA). Responders indicate the frequency they agree with statements such as ‘I feel emotionally drained from my work’ and ‘I don’t really care what happens to some patients.’[40] By this scale, an individual is suffering from symptoms of burnout if they exhibit a high EE score or a high DP score; an individual may also be suffering from burnout if they exhibit a high EE score plus either a high DP score *or* a low PA score The prevalence of burnout in the medical population exceeds 67% and it is associated with impaired clinical decision making, malpractice, professionalism lapses, and adverse personal outcomes including substance use and depression [41] The MBI is recognized by the National Academy of Medicine as one of the most frequently used scale to measure burnout[42]. One 2019 visual arts study utilized the MBI scale to study the effects of an arts intervention on internal medicine residents[34].

## COMMUNICATION

### Communication Skills and Attitude Test

The Communication Skills and Attitude Test (CSAS) was developed in 2002 by UK psychiatrist Charlotte Rees for measuring medical students’ attitudes towards learning communication skills[43]. The 26-item questionnaire is scored on a Likert Scale and is predicated on the belief that to be an effective healthcare practitioner, effective communication and an openness to improve one’s communication skills are paramount. Example statements include, ‘In order to be a good doctor I must have good communication skills’ and ‘I find it hard to admit to having some problems with my communication skills.’[43] The CSAS has been utilized in two visual art and medical education studies [31,32].

## EMPATHY & COMPASSION

### The Compassion Scale

The Compassion Scale (CS) was developed in 2011 by psychologist Elizabeth Pommier at the University of Texas at Austin to measure an individual’s understanding of and response to the suffering of others [44,45]. The 24-item questionnaire is scored on a Likert scale. It builds upon psychologist and co-creator Kristin Neff’s model of self-compassion and measures compassionate responses through three conflicting domains: kindness-versus-indifference, common humanity-versus-separation, and mindfulness-versus-disengagement[44].

*Kindness* is defined as concern for those who are suffering linked with a desire to console. It is assessed using statements such as ‘If I see someone going through a difficult time, I try to be caring towards that person.’[44] Its antithesis, *indifference*, is assessed using statements such as ‘I don’t concern myself with other people’s problems.’[44] *Common humanity* is broadly defined as one’s ability to recognize that all humans suffer and simultaneously feel a sense of connection to those who are suffering. It is assessed using statements such as ‘Despite my differences with others, I know that everyone feels pain just like me.’[44] Its antithesis, *separation*, attempts to capture isolation through statements such as ‘I can’t really connect with other people when they’re suffering.’[44] *Mindfulness* is defined as ‘balanced awareness that neither avoids nor gets lost in others pain’ coupled with a desire to make oneself aware of the other’s suffering[44]. Its antithesis, *disengagement*, is an obliviousness to suffering coupled with a lack of desire to offer consolation. Disengagement is measured with statements such as ‘I try to avoid people who are experiencing a lot of pain.’ [44,45] The scale has been utilized in one visual arts and medical education study[36].

### The Interpersonal Reactivity Index

The Interpersonal Reactivity Index (IRI) was developed in 1980 by psychologist Mark Davis at Eckerd College to measure cognitive and affective empathy [46,47]. The 28-item questionnaire is scored on a Likert Scale and measures empathy along four distinct subdomains. *Cognitive empathy* is assessed by examining perspective-taking and fantasy. The perspective-taking statements measure an individual’s ability to assume the psychological point of view of others while the fantasy statements examine an individual’s ability to imagine themselves as agents in fictional narratives. *Affective empathy* is assessed by examining empathic concern and personal distress. Empathic concern statements evaluate an individual’s ability to feel sympathy towards someone who is suffering, while the personal distress statements evaluate an individual’s emotional distress that results from witnessing suffering. A shortened version of the IRI has been included in the AAMC Matriculating Student Questionnaire and the Graduating Questionnaire. The IRI has been used in two visual arts-based studies [35,36].

### Jefferson Scale of Empathy

The Jefferson Scale of Empathy (JSE) was developed in 2007 by Mohammadreza Hojat, a research professor of psychiatry at Jefferson Medical College, to measure cognitive and affective empathy[48]. The 20-item questionnaire is scored on a Likert Scale and it assesses three components of empathy: perspective taking, compassionate care, and the ‘ability to stand in patients’ shoes.’[48] The scale is available in 56 different languages and includes modifications for medical students, health professionals, and health professional students. Example statements in the medical student version include, ‘patients feel better when their physicians understand their feelings.’[7] This scale has been utilized in one visual arts and medical education study[24].

### Reading the Mind in the Eyes

The Reading the Mind in the Eyes test was designed in 1997 by psychologist Simon-Baron Cohen at Cambridge University in the UK to study emotional recognition in patients with Asperger syndrome or high-functioning autism [49–51]. The test is based upon Theory of Mind psychology, which ‘is the ability to recognize the thinking or feelings of others in order to predict their behaviors and act accordingly.’[51] The test asks individuals to match an emotion with images of peoples’ eyes. It is grouped with empathy because the recognition of others’ emotional state is the first step in acting empathetically. Jaclyn Gurwin, an ophthalmologist at the University of Pennsylvania, explored students’ emotional recognition abilities as a secondary outcome with the Reading the Mind in the Eyes test in a visual arts intervention designed to improve first year medical students’ ability to describe retinal and periorbital pathology[28].

## GRIT

### The Short Grit Scale

The Short Grit Scale (Grit-S) was created in 2009 by Angela Duckworth, a psychologist at the University of Pennsylvania, to measure grit, which is the innate desire to pursue and achieve long-term goals regardless of external positive reinforcement[52]. The 8-item questionnaire is scored on a Likert scale and evaluates grit along two domains: ‘consistency of interest’ and ‘perseverance of effort.’[52] Consistency of interest is assessed using statements such as ‘I have been obsessed with a certain idea or project for a short time but later lost interest.’[52] Perseverance of effort is assessed with statements such as ‘I finish whatever I begin.’[52] This grit scale has been used in one visual arts and medical education study[24].

## MINDFULNESS & REFLECTION

### The Five-Facet Mindfulness Scale

The Five-Facet Mindfulness Scale was designed in 2006 by University of Kentucky psychologist Ruth Baier to assess mindfulness[53]. The 39-item questionnaire is scored on a Likert Scale and evaluates five distinct domains: observing, describing/self-expression, acting with awareness, non-judgment of inner experience, and non-reactivity to inner experience[53]. *Observing* is assessed using statements such as ‘I pay attention to how my emotions affect my thoughts and behavior.’[53] *Description* and *self-expression* are assessed using statements such as ‘I can usually describe how I feel at the moment in considerable detail.’[53] *Acts of self-awareness* are assessed using statements such as ‘when I do things, my mind wanders off and I’m easily distracted.’[53] The *non-judgment of inner experience* is assessed using statements such as ‘I criticize myself for having irrational or inappropriate emotions’[53] Lastly, *non-reactivity to inner experience* is assessed with statements such as ‘I perceive my feelings and emotions without having to react to them.’[53] The scale was utilized in one visual arts and medical education study[36].

### The Groningen Reflective Ability Scale

The Groningen Reflective Ability Scale (GRAS) was developed in 2009 at the University of Groningen in the Netherlands by Leo Aukes, a researcher at the Center for Research and Innovation of Medical Education, and Joris Slaets, a physician and Professor of Geriatrics[54]. The 23-item questionnaire is scored on a Likert Scale and measures a medical professional’s reflective ability. Predicated on the belief that reflection is required for maintenance of professional competence and personal wellbeing, the questionnaire evaluates three domains: self-reflection, empathic reflection, and reflective communication.

Self-reflection statements explore an individual’s ability to engage in ‘introspection, exploration, understanding, and appraisal of experiences.’[54] Empathic reflection statements examine the ability to intuit others’ experiences. Reflective communication statements assess one’s openness for feedback and one’s willingness to accept accountability for their actions. The GRAS was utilized in one visual art and medical education study[27].

### The Mindful Attention Awareness Scale

The Mindful Attention Awareness Scale (MAAS) was developed in 2003 by University of Rochester psychologists Richard Ryan and Kirk Brown to assesses dispositional mindfulness, which is the ability to be aware of and pay attention to the present moment[55]. Brown and Ryan reinforce that mindfulness is a measure of consciousness distinct from other forms of mental processing, such as cognition or emotion. Consciousness, and by extension mindfulness, requires an *awareness* of the inner and outer environment coupled with a focused *attention* of one’s conscious mind. The 15-item questionnaire is scored on a Likert Scale and it includes statements such as ‘I tend not to notice feelings of physical tension or discomfort until they really grab my attention.’[55] The MAAS was used in a visual arts and medicine study of third year medical students[24].

## Discussion: Putting the Toolkit to Use

We surveyed studies of visual art programming and identified several de-novo quantitative scales used to assess observation skill and twelve psychometric scales used to assess a variety of domains: tolerance for ambiguity, bias, burnout, communication, empathy, grit, and mindfulness/reflection (Table 1, Table 2). Some psychometric scales originated in the general psychology literature while others were developed specifically for clinical care and education. Some scales, such as the Jefferson Scale for Empathy and Geller’s modified Tolerance for Ambiguity scale have been widely adopted; others, such as the Best Intentions Questionnaire and Reading the Mind in the Eyes test, have gained little traction. The variability of scales used to assess the impact of visual art programming reflects, in part, a lack of consensus regarding how best to measure the utility of visual art interventions for medical trainees. It may also reflect a limited awareness of the universe of available tools with which to evaluate such programming. We hope that this narrative review will make educators planning to evaluate the impacts of visual arts programming aware of the broader universe of analytic tools in order that they can choose the most appropriate one.

Most clinicians and healthcare educators would agree that these metrics attempt to measure domains relevant to medical practice. But many published educational studies do not evidence a clear congruence between their curricular design and the metrics chosen to measure their outcome. It often seems that these scales are indiscriminately incorporated into studies based on ease of administration and interpretation, rather than being carefully selected to match targeted curricular interventions. This practice may also occur, in part, because of a belief that most visual art-based methodologies equally address all the aforementioned domains [12,13,15]. This imprecision has limited our ability to understand the effects of visual arts programming.

Prior to selecting any psychometric tool, study designers should first explore the strengths and weakness of that tool. Consider the psychometric scales used to measure cognitive empathy. In recent years, researchers have attempted to demonstrate quantitatively that empathy changes, and in many cases declines, throughout medical training [7,9–11]. But some argue that the measurement scales used to assess empathy may be flawed given the lack of an agreed-upon operational definition of empathy, the lack of inter-instrument reliability, and, perhaps most important, their reliance on student (or physician) self-assessment [56–61]. Given that ‘the preponderance of evidence suggests that physicians have a limited ability to self-assess,’ would it not be more valuable to evaluate empathy through a third-party assessment of clinical encounters with patients (either real or standardized)? [60–62] All of the psychometric scales cited in this review also require individuals to internally judge their agreement (or disagreement) with various statements related to ambiguity, bias, burnout, compassion, communication, and grit. Educators wishing to evaluate novel visual arts programming using psychometric measurement should take this locus of measurement into account before selecting one of these psychometric scales.

How, then, might one use this toolkit? First, educators should attempt to align psychometric scales or quantitative observation metrics with *specific* educational goals. These goals could include one (or more) of the learning objectives outlined in the AAMC-endorsed Prism model: Mastering Skills, Perspective Taking, Personal Insight, and Social Advocacy[20]. For example, an educator who wants to address the ‘Personal Insight’ domain using a *targeted* visual arts-based intervention may wish to use the psychometric scales that assess tolerance of ambiguity (modified TFA), burnout (MBI), empathy (JSE, IRI), and mindfulness (MAAS). Other psychometric scales not yet utilized in the visual arts and medical education literature may also be useful. For example, those endorsed by the National Academy of Medicine may be especially useful to measure burnout and healthcare professional well-being.^42^

Second, educators should be aware of the pitfalls of psychometric measurement based on learner self-assessment. Educators may want to pair psychometric measurement with additional data (e.g., qualitative interview, third-party assessment) to evaluate novel curricula more holistically. Finally, educators should consider using these psychometric tools for long-term, longitudinal analysis. While pre-test/post-test measurements may be helpful to gauge the short-term impacts of a visual arts-based curriculum, long-term outcomes related to burnout, professionalism, healthcare worker wellbeing, patient-level outcomes, and other underexplored domains among visual arts programming participants will likely provide much more meaningful data[23]. Carefully matching curricular methodology with psychometric scales, third party assessment, and qualitative data, when accompanied with long term follow-up and reassessment, will build a more cohesive body of medical humanities literature that promotes inter-institution scalability of visual arts programming.

## Conclusion

Novel visual arts and humanities pedagogies may need to be formally evaluated to gain recognition and approval. The NASEM report when coupled with the AAMC monograph and Prism model can support efforts to integrate and disseminate the visual arts into medical education curricula nationwide. This narrative review of measurement scales provides educators with a toolkit of resources to design and evaluate visual arts and medical education initiatives.
